# An fMRI investigation of the effects of attempted naming on word retrieval in aphasia

**DOI:** 10.3389/fnhum.2015.00291

**Published:** 2015-05-26

**Authors:** Shiree Heath, Katie L. McMahon, Lyndsey A. Nickels, Anthony Angwin, Anna D. MacDonald, Sophia van Hees, Eril McKinnon, Kori Johnson, David A. Copland

**Affiliations:** ^1^ARC Centre of Excellence in Cognition and its Disorders, Department of Cognitive Science, Macquarie University, SydneyNSW, Australia; ^2^Language Neuroscience Laboratory, University of Queensland Centre for Clinical Research, St LuciaQLD, Australia; ^3^Centre for Advanced Imaging, University of Queensland, St LuciaQLD, Australia; ^4^NHMRC Centre for Clinical Research Excellence in Aphasia Rehabilitation, St LuciaQLD, Australia; ^5^School of Health and Rehabilitation Sciences, University of Queensland, St LuciaQLD, Australia

**Keywords:** aphasia, repeated picture naming, word retrieval, fMRI

## Abstract

In healthy controls, picture naming performance can be facilitated by a single prior exposure to the same picture (“priming”). This priming phenomenon is utilized in the treatment of aphasia, which often includes repeated picture naming as part of a therapeutic task. The current study sought to determine whether single and/or multiple exposures facilitate subsequent naming in aphasia and whether such facilitatory effects act through normal priming mechanisms. A functional magnetic resonance imaging paradigm was employed to explore the beneficial effects of attempted naming in two individuals with aphasia and a control group. The timing and number of prior exposures was manipulated, with investigation of both short-term effects (single prior exposure over a period of minutes) and long-term effects (multiple presentations over a period of days). Following attempted naming, both short-term and long-term facilitated items showed improvement for controls, while only the long-term condition showed benefits at a behavioral level for the participants with aphasia. At a neural level, effects of long-term facilitation were noted in the left precuneus for one participant with aphasia, a result also identified for the equivalent contrast in controls. It appears that multiple attempts are required to improve naming performance in the presence of anomia and that for some individuals with aphasia the source of facilitation may be similar to unimpaired mechanisms engaged outside the language network.

## Introduction

Aphasia is a chronic condition and word retrieval impairments are prevalent in most people with aphasia (Goodglass and Wingfield, 1997; Damasio, 2001). Consequently, it is essential to understand the mechanisms underlying word retrieval that can be capitalized on to maximize recovery. There is clear evidence that treatment for word retrieval impairments can be effective (for review see Nickels, 2002a,b), and emerging understanding of the neural mechanisms underlying these treatment effects (Meinzer et al., 2011; Heath et al., 2012, 2013; van Hees et al., 2014a,b). However, there remains much to be learned in order to ensure optimal recovery for people with aphasia. One of the most exciting possibilities is the finding that improvements in word retrieval can occur with no direct treatment and well past the period when spontaneous recovery is expected (Nickels, 2002a,b). Even a single attempt at naming a picture has been found to improve subsequent retrieval and naming of the same item in some people with aphasia (Howard, 2000; Nickels, 2002a,b). For such individuals, this finding suggests that naming ability may be improved simply through attempting to retrieve the words they wish to say. However, there has been little research to examine the nature of the cognitive and neural processes underlying this improvement and the conditions under which they occur. This paper aims to do just that: we focus on two individuals with chronic aphasia and examine the behavioral and neural effects of prior attempts at naming on subsequent naming.

The positive effect of performing one task on a subsequent task is termed “facilitation” (Patterson et al., 1983), or “repetition priming” in unimpaired speakers (Tulving and Schacter, 1990; Cave, 1997; Gotts et al., 2012; Henson et al., 2014). For picture naming, positive effects are evidenced by increased naming accuracy for individuals with aphasia and decreased naming latencies for unimpaired speakers. Studies exploring the neurocognitive mechanisms underlying the priming of picture naming in unimpaired speakers (van Turennout et al., 2000, 2003; Meister et al., 2005; MacDonald et al., under review) have found reduced activation (“repetition suppression”) in visual and language-related cortical regions for repeated stimuli, which is thought to be an indication of greater processing efficiency for previously seen items (Gotts et al., 2012). Increases in activity (“repetition enhancement”) have also been found, but these are often associated with some form of additional processing required upon repeated stimulus presentation (Henson et al., 2000; Henson, 2003). For repetition of the same stimuli, increases in activity may be due to the additional processing related to stimulus recognition, explicit memory retrieval, expectation, or attention (Segaert et al., 2013).

Several explanations for the phenomenon of repetition priming have been put forward. One account holds that priming is mediated by episodic retrieval (Mitchell and Brown, 1988; Tenpenny, 1995), suggesting that a memory trace is generated on prime presentation and its subsequent ease of retrieval is the source of facilitation. Another proposal suggests that priming involves a modification of stored cognitive representations during first presentation, resulting in enhanced recognition and/or retrieval processes, or a lowering of activation thresholds, upon subsequent presentation (Morton, 1969; Wheeldon and Monsell, 1992). In other words, a repeated stimulus is responded to faster and/or more accurately because it engages the same, now primed, processing, and representations (Barry et al., 2001).

While the exact cognitive bases of facilitation effects remain undetermined, previous research has indicated that effective word finding treatments may be acting through these ‘normal’ priming mechanisms. Evidence supporting this argument is provided by research investigating priming effects in individuals with aphasia using a different facilitating task. For example, Hickin et al. (2002) explored the association between response to phonological cues and improved naming ability in eight individuals with aphasia following phonological treatment. The authors identified a significant correlation between positive response to facilitation and positive response to treatment, highlighting the utility of facilitation as a possible predictor of treatment outcome. It is therefore important to understand the neural processing underlying such positive effects and consider any impact this improved understanding may have for rehabilitation. The current study aimed to conduct an fMRI investigation into the facilitatory effects of repeated picture naming in individuals with aphasia and healthy older adults. The experiment manipulated the timing and number of prior naming attempts to examine short-term (from one single attempt minutes prior to subsequent naming of the same item) and long-term (from multiple naming attempts of the same item over several days) effects.

While research exploring facilitation from attempted naming has generally been limited to behavioral studies (Nickels, 2002a), some research has investigated the brain regions engaged during accurate, and inaccurate picture naming by individuals with aphasia (Fridriksson et al., 2009; Postman-Caucheteux et al., 2010; Sebastian and Kiran, 2011). These studies can be informative regarding the neural correlates of specific types of linguistic processing, but they do not provide insights into training-induced effects or their longevity. Other neuroimaging studies have looked at the effects of naming treatments (Fridriksson et al., 2006b; Menke et al., 2009; Kiran et al., 2013), however, these often utilize a pre-post paradigm which can be confounded by differences in performance, with neural activation during impaired pre-treatment performance compared to improved performance following treatment (Poldrack, 2000).

The current investigation sought to advance our understanding of the brain-behavior relationship involved in improvement of word retrieval ability. A picture naming task was administered within a single fMRI scanning session, with only accurately named responses analyzed. Analyses of correct responses focused our investigation on the positive effects brought about by repeated picture naming, as might be seen following treatment, and therefore maximized the therapeutic implications of the study. Furthermore, previous research has shown differential patterns of brain activity for accurate word retrieval versus error responses (Meinzer et al., 2006; Fridriksson et al., 2009; Postman-Caucheteux et al., 2010). Any averaged results across all naming trials irrespective of response type would therefore be difficult to interpret with regard to the neural substrates of successful word retrieval, as results may also include activity reflecting error processing and/or increased effort during incorrect naming. In this study, both the timing and number of naming exposures was manipulated across critical conditions, allowing comparison of accurately named items that had not previously been facilitated to items that had received one or more earlier naming attempts. It was hypothesized that if the priming mechanisms at work in unimpaired speakers do indeed underlie effective naming treatment in individuals with aphasia, then repetition suppression effects in similar regions for short-term and long-term conditions may be evident across controls and aphasic participants. As picture naming involves all word production processes, it was proposed that modulation of activity would be identified in a combination of regions known to be involved in both semantic (Démonet et al., 1992, 2005; Binder et al., 1997, 2009; Bookheimer, 2002; Abrahams et al., 2003; Vigneau et al., 2006) and phonological-type (Moore and Price, 1999; Bookheimer, 2002; Abrahams et al., 2003; Hickok and Poeppel, 2004; Indefrey and Levelt, 2004; Vigneau et al., 2006) processes. For participants with aphasia, this could include spared left hemisphere regions associated with word production, or areas close to damaged language-related regions and/or in the right hemisphere homologues of these regions. It was further hypothesized that, in line with evidence from previous behavioral research in unimpaired speakers and in aphasia (Cave, 1997; Nickels, 2002a), a single prior exposure could effectively facilitate naming performance in participants with aphasia.

## Materials and Methods

### Participants

**FIGURE 1:**
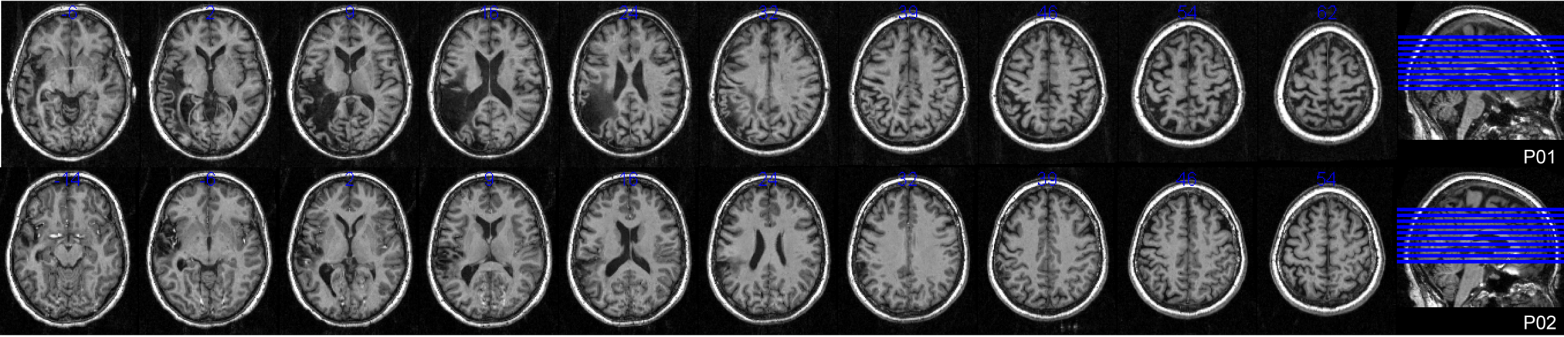
**Lesion locations.** Axial T_1_ weighted MRI slices at 5 mm intervals for both participants with aphasia.

Ethical approval was obtained from the University of Queensland and all participants gave informed written consent under an approved Medical Research Ethical Review Committee protocol. Eighteen control participants (seven male) meeting inclusionary criteria participated in the study. Their average age was 54.4 years (SD 8.8) and educational level 16.1 years (SD 3.6). Full details of the experiment conducted with controls are reported elsewhere (MacDonald et al., under review). Two female participants with aphasia were also recruited. Both the Western Aphasia Battery (WAB; Kertesz, 1982) and the Comprehensive Aphasia Test (CAT; Swinburn et al., 2004) were used to determine the presence and classification of aphasia. The full assessment battery also included the Boston Naming Test (BNT; Kaplan et al., 1983) and the Pyramids and Palm Trees Test (P and PT; Howard and Patterson, 1992). **Table 1** provides full demographic, clinical, and assessment battery details, as well as the possible levels of impairment in spoken word production for both participants. **Figure 1** displays lesion location, which for both individuals primarily included temporo-parietal regions. All participants reported English as their first language, were right handed, and had normal (or corrected to normal) vision. Every participant was screened for cognitive impairment by administration of the Mini-Mental State Examination (Folstein et al., 1975) and for depression using the Geriatric Depression Scale (Sheikh and Yesavage, 1986).

**Table 1:** Demographic, clinical, and assessment battery results for participants with aphasia.

	Max	P01	P02
**Gender:**		F	F
**Age:**		71	59
**Level of education:**		10	10
**Time post-stroke:**		14 years; 1 month	2 years; 4 months
**Lesion volume (cm^3^):**		83.13	22.68
**Lesion involvement:**		•Supramarginal gyrus • Angular gyrus • Superior temporal gyrus • Middle temporal gyrus • Heschl’s gyrus • Rolandic operculum • Insula • Superior occipital gyrus • Middle occipital gyrus	•Supramarginal gyrus • Superior temporal gyrus • Middle temporal gyrus • Hippocampus
**Naming pre-tests (*n* = 952):**
- Correct		49.5%	53.0%
- Error breakdown:			
∘ Phonological errors		13.11%	25.94%
∘ Semantic errors		15.41%	9.62%
∘ Do not know/no response		62.87%	57.72%
∘ Other errors		8.61%	6.72%
**WAB:**
- Spontaneous speech - Comprehension - Repetition - Naming/word finding - Aphasia quotient - Classification	20 200 100 100 100	14 111 68 71 66.8^∗^ Wernicke’s	17 166 46 85 76.8^∗^ Conduction
**BNT:**	60	24^∗^	33^∗^
**P and PT:**
- Three pictures - Spoken word, two pictures	52 52	45^∗^ 45^∗^	51 48^∗^
**CAT:**
- Spoken comprehension - Repetition (five subtests): ∘ Real words ∘ Complex words ∘ Non-words - Naming - Fluency - Reading - Spoken picture description	66 74 32 6 10 58 un 70 un	48* 43* 22 3 6 49* 16 59* 19*	48* 26* 17 1 0 46* 20 57* 40
**Possible levels of impairment in spoken word production:**		•Semantics •Semantics to phonology and/or phonological form	•Semantics to phonology and/or phonological form •Post-phonology

Max, maximum raw score possible; un, unlimited possible maximum score. ¤ error percentages include self-corrections. ^∗^Indicates score falls below normal range with reference to available normative data for the Western Aphasia Battery (WAB; Kertesz, 1982), the Pyramids and Palm Trees (P and PT; Howard and Patterson, 1992), the Comprehensive Aphasia Test (CAT; Swinburn et al., 2004), and in the case of the Boston Naming Test (BNT) with reference to Australian normative data (Cruice et al., 2000). Lesions were identified using an automated method (Stamatakis and Tyler, 2005) and volumes calculated using fslstats (FMRIB Software Library – www.fmrib.ox.ac.uk/fsl).

### Stimuli and Procedure

A large collection of 476 similarly sized gray-scaled picture stimuli was obtained (Hemera Photo-Objects, Hemera, Hull, QC, Canada and royalty free digital stock photographs). For controls 180 of these pictures were then divided into nine sets of 20 items, with assignment of one set to the short-term facilitated condition and one set to the long-term facilitated condition (counterbalanced across participants). For participants with aphasia two pre-test sessions were conducted, in which the entire collection was presented at each session in a random order for naming. Based on the results of both pre-test sessions, 60 pictures were then chosen as experimental stimuli and divided into three sets of 20 items. Stimuli for the short-term facilitated and long-term facilitated conditions were sourced from those items participants found difficult to name across both pre-test sessions and therefore represented suitable targets for facilitation. A third set of stimuli formed an unfacilitated condition, chosen from items that were named consistently by participants during pre-test sessions. It should be noted that P01 was unable to name most items during both pre-tests. However, P02 was more inconsistent, with the majority of items inaccurately named or not responded to in at least one pre-test.

All sets were matched on the basis of International Picture Naming Project naming reaction time (Szekely et al., 2004), frequency (combined written and spoken; Baayen et al., 1995), number of phonemes, number of syllables, and percentage name agreement (Szekely et al., 2004). Additionally, control sets were matched for age of acquisition (Morrison et al., 1997) and imageability (Wilson, 1988). Following the pre-test sessions, a facilitation phase and an experimental phase were conducted. The facilitation phase required all participants to complete two sessions within 2–3 days, during which only the long-term facilitated set of stimuli was presented, in random order, three times for (attempted) overt naming (six times in total over the two sessions). A single facilitation trial consisted of a fixation cross (1.5 s), followed by display of the target picture (3 s). No feedback was provided on performance, nor were any cues provided. The experimental phase was conducted ∼2 days after completion of the facilitation phase.

During the experimental phase, all sets of stimuli were presented for naming during an fMRI scanning session. A single trial lasted 14.7 s and involved presentation of a fixation cross (2 s), blank screen (250 ms), target picture (3 s), and blank screen (9.45 s). The photographic stimuli were back-projected onto a screen viewed through a mirror mounted on the head coil (10° of visual arc). Naming responses were digitally recorded (sampling rate 11 kHz) with an optical single channel noise-canceling microphone (FOMRI, Optoacoustics Ltd., Or-Yehuda, Israel). The items presented in the previous facilitation sessions were presented again in the scanner to investigate any long-term effects from previous naming attempts. The short-term facilitation set was presented twice within the scanner: the first presentation and naming attempt served as a potential prime and the second as a target, with an average lag of 7.4 trials (range: 6–10) between the two presentations, to investigate any short-term facilitation effects. For all participants, the initial short-term prime presentations represented an unfacilitated condition.

**FIGURE 2:**
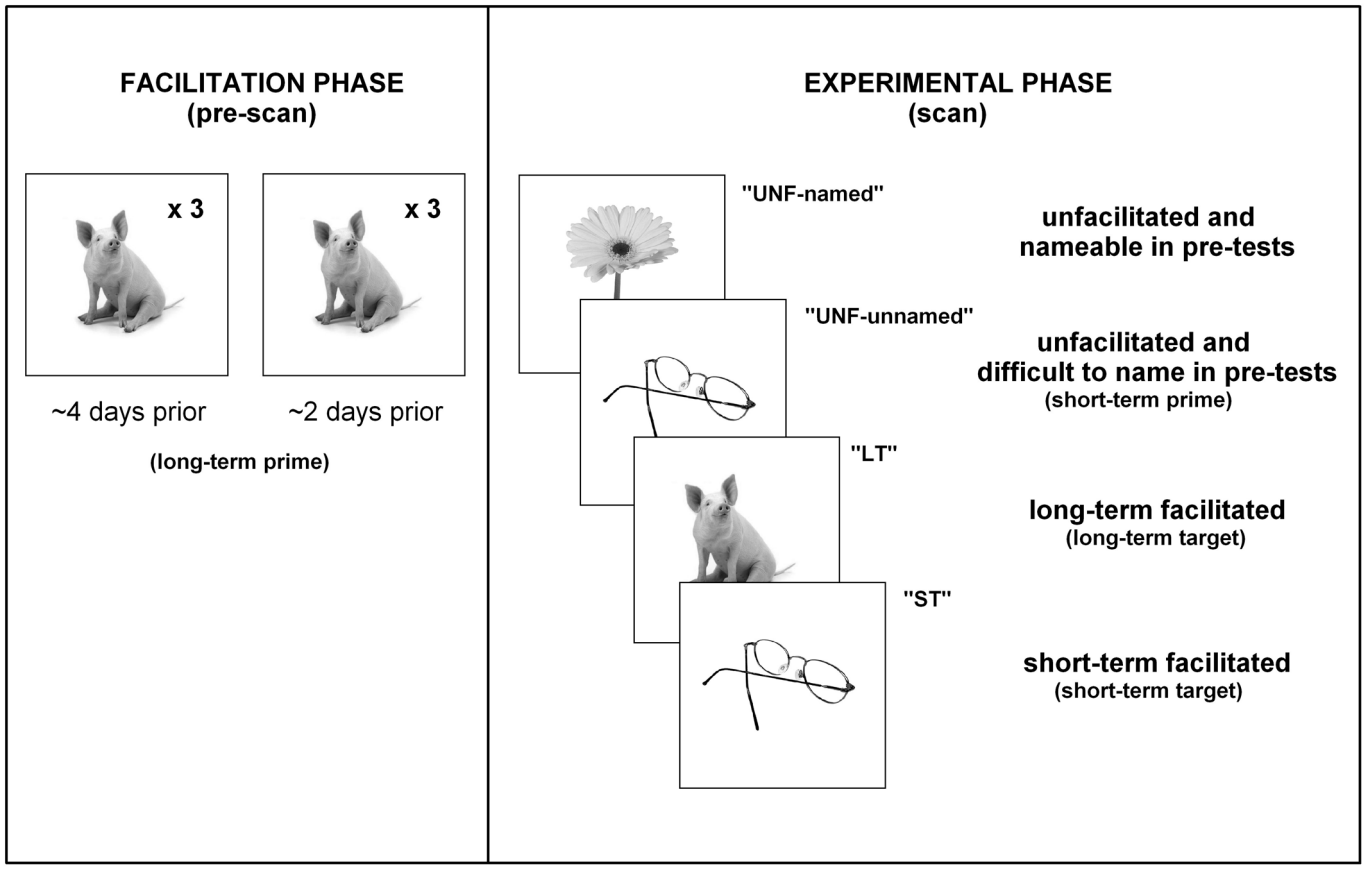
**Procedures.** A summary of the presentation of stimuli/conditions for participants with aphasia.

Therefore, the three main conditions of interest presented in the scanner for controls were unprimed (short-term prime), short-term facilitated (short-term target) and long-term facilitated (long-term target). For participants with aphasia, the four main conditions were: unfacilitated items that were nameable during pre-test (“unfacilitated-named”), unfacilitated items that were difficult to name during pre-test (“unfacilitated- unnamed” short-term prime presentations), short-term facilitated items difficult to name (“short-term” target presentations), and long-term facilitated items difficult to name (“long-term”). Refer to **Figure 2** for a breakdown of conditions and procedures for participants with aphasia.

### Processing and Analyses

**FIGURE 3:**
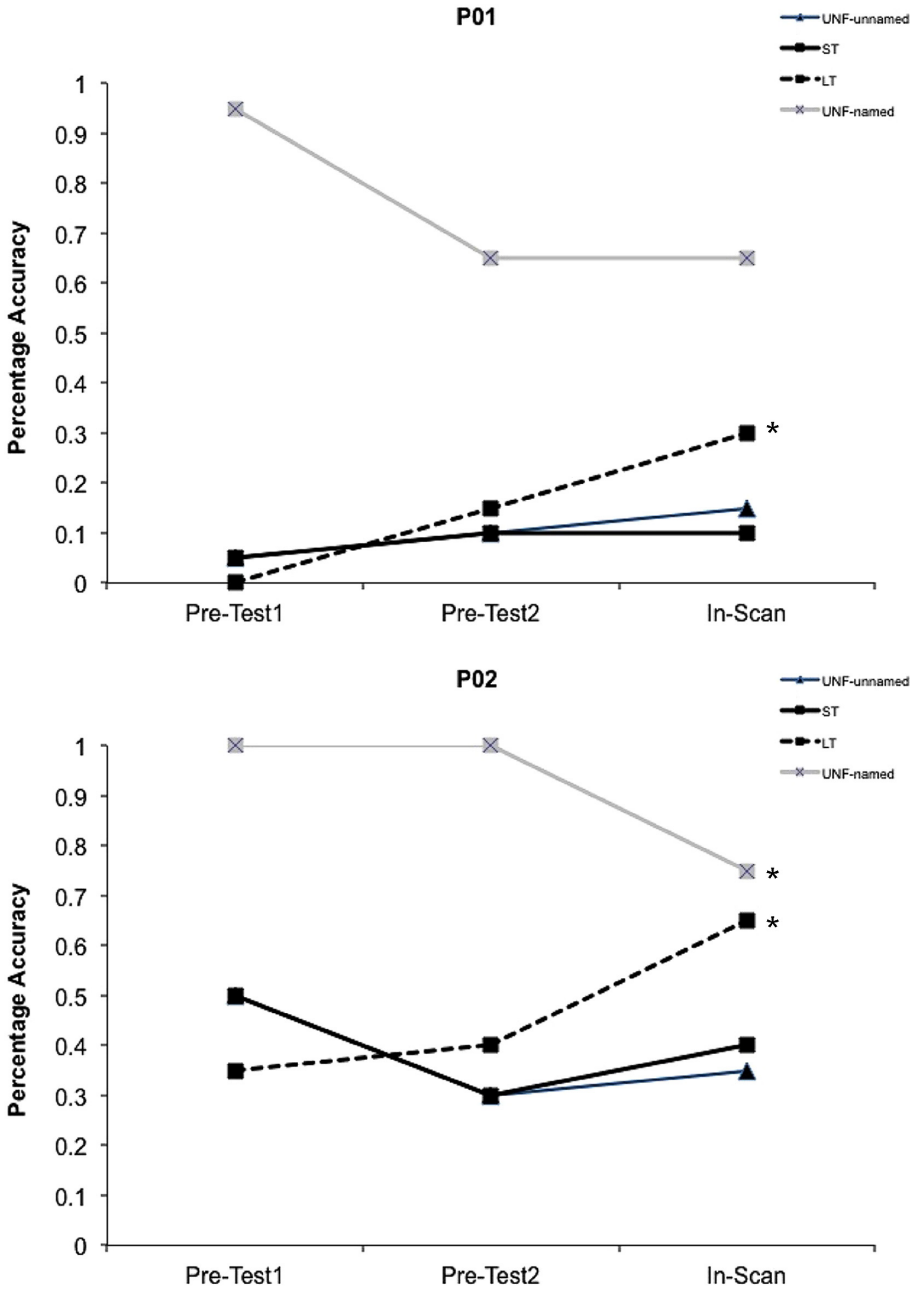
**Behavioral data.** Facilitation effects in percentage accuracy data for both participants with aphasia for all conditions. The initial short-term prime presentations within the scanner (In-Scan) represent the UNF-unnamed condition. ^∗^Indicates a significant difference (*p* < 0.05) between pre-facilitation and post-facilitation percentage accuracy.

During each session a high resolution (1.0 mm × 1.0 mm × 1.0 mm) T_1_ weighted magnetization-prepared rapid gradient-echo structural scan was acquired (repetition time 2200 ms, echo time 2.99 ms, flip angle 9°). BOLD imaging with gradient-echo, echo planar image (GE-EPI) acquisition was conducted (matrix size 64 × 64; repetition time 2100 ms; echo time 30 ms; 90° flip angle; field of view 230 mm), with a total of 480 GE-EPI volumes acquired over two runs at 4-Tesla (the first five volumes of each run were discarded). Images were acquired in 36 axial planes with an in-plane resolution of 3.6 mm and slice thickness of 3 mm (0.6 mm gap). A behavioral interleaved gradient design was utilized to avoid the artifacts associated with head movement during overt speech, as well as to enable recording of responses and reaction times. To minimize scanner noise during picture presentation and response periods (4.2 s), only slice gradients were applied during the critical interval with radiofrequency intact to maintain steady state magnetization (Eden et al., 1999). Image acquisition for each trial therefore occurred during the remaining 10.5 s (blank screen and fixation cross) so as to capture the blood oxygen level-dependent (BOLD) response. A point-spread function mapping sequence was acquired prior to GE-EPI acquisitions, allowing the distortion in geometry and intensity to be corrected in the time series data. Target trials that elicited no response or an incorrect response from participants were excluded from the behavioral and imaging analyses. For the two participants with aphasia, the number of correct trials for each condition within the scanner were: P01 – unfacilitated-unnamed 3, short-term 2, long-term 6, unfacilitated-named 13; P02 – unfacilitated-unnamed 7, short-term 8, long-term 13, unfacilitated-named 15 (refer to **Figure 3**).

Images were processed using statistical parametric mapping software (SPM5^1^). The image time series were realigned using INRIAlign rigid body motion correction (Freire et al., 2002). For each participant the mean EPI was then co-registered with their T_1_ image, followed by segmentation and normalization to the standard Montreal Neurological Institute (MNI) template (Evans et al., 1993). Spatial smoothing (8 mm full-width half-maximum Gaussian kernel) was applied to normalized volumes (3.0 mm × 3.0 mm × 3.0 mm). A general linear model (GLM) was constructed for the fMRI time series using finite impulse response functions (due to the behavioral interleaved design) resulting in partial collection of hemodynamic response, with onsets and durations chosen to reflect the expected peak BOLD response. Group level whole brain analyses were conducted for controls, with results reported for clusters greater than 20 voxels (*p* < 0.05 FDR corrected, with age included as a covariate in the GLM). Single subject whole brain analyses for the participants with aphasia were conducted. To correct for multiple comparisons 3dClustSim (Analysis of Functional Neuroimages, National Institute of Mental Health, Bethseda, MD, USA) was used, inputting the full-width at half maximum (FWHM) of noise calculated from the square root of the residuals using 3dFWHMx. By adopting a height threshold of *p* < 0.001, a family wise error rate of *p* < 0.05 was achieved with a minimum cluster threshold of 86 voxels for P01 and 55 voxels for P02.

## Results

### Control Participants

Results for control participants are reported separately (MacDonald et al., under review). Briefly, the behavioral results showed significant priming effects for both long-term and short-term facilitated conditions. The whole brain results identified a relative decrease of activation for long-term facilitated items when compared to unfacilitated items (comparable to the unfacilitated-named condition in participants with aphasia) in the bilateral pars triangularis of the inferior frontal gyri (IFG), in the bilateral posterior inferior temporal gyri, and in the right precentral gyrus. Greater activation, however, was found for facilitated items than for unprimed items in several regions: in the bilateral precuneus for long-term facilitated items, and in the right precuneus and the right middle frontal gyrus for short-term facilitated items.

### Participants with Aphasia

Percentage accuracy data for the pre-test sessions and within the scanner are shown in **Figure 3** for both participants. A weighted Wilcoxon One-Sample test was used to determine whether accuracy differed from pre-facilitation (Pre-Tests 1 and 2) to post-facilitation (In-Scan) for each condition. For the unfacilitated stimuli that were previously able to be named, as expected under the somewhat stressful conditions of the scanner and consistent with regression to the mean, performance decreased during the scanning session, significantly in the case of P02 (*p* = 0.01). Both P01 and P02 showed a significant positive change in accuracy following long-term facilitation (*p* < 0.02), but not in the short-term, or unfacilitated-unnamed conditions.

A Mann–Whitney *U*-test was also subsequently conducted to determine whether the magnitude of change from pre-to post-facilitation differed between specific conditions. A significant difference was identified for P02 between the long-term and unfacilitated-unnamed conditions (*p* = 0.005). However, there were no significant differences for either participant between short-term and unfacilitated-unnamed, or between short-term and long-term conditions. An analysis of reaction times was not conducted for the participants with aphasia.

Full whole brain neuroimaging results for participants with aphasia are set out in Supplementary Table S1, however, we report and discuss only those results for contrasts involving two conditions, the long-term and unfacilitated-named conditions. This is due to the fact that we did not find significant behavioral results for the short-term or unfacilitated-unnamed conditions and these two conditions also had very few trials in which an accurate response was produced within the scanner.

**FIGURE 4:**
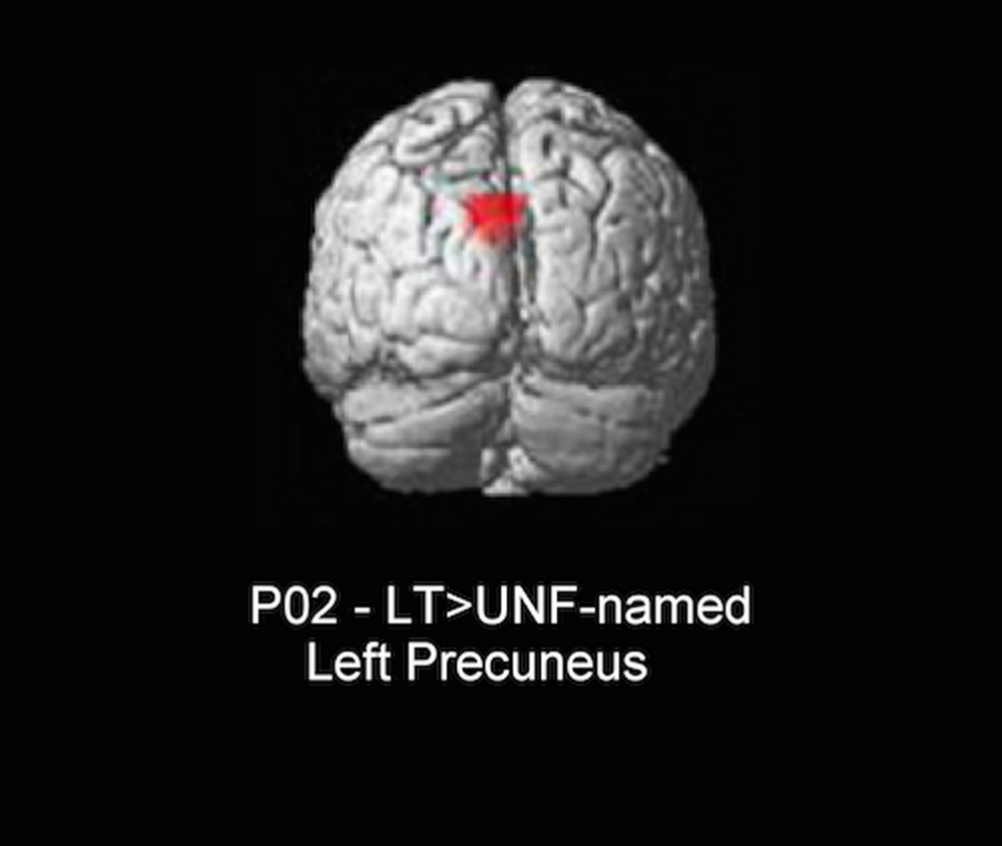
**Whole brain result for P02 (3dClustSim minimum cluster threshold 55 voxels).** LT, long-term facilitated and previously difficult to name; UNF-named, unfacilitated and previously accurately named.

The only significant difference in neural activity was found for P02, with greater activity for long-term facilitated items (facilitated and previously difficult to name) when compared to unfacilitated-named (unfacilitated and previously accurately named) items in the left precuneus (see **Figure 4**). In controls, the same precuneus region was identified for the equivalent contrast. Further, as we found long-term results in the whole brain analyses within the left inferior frontal region for controls, a subsequent bilateral region of interest (ROI) analysis in this area was conducted for both individuals with aphasia. Anatomical ROIs were created with the WFU_PickAtlas^2^ toolbox within SPM5, using the IBASPM116 atlas and implementing WFU ROI masking (Maldjian et al., 2003, 2004). Six ROIs were analyzed for each participant, including bilateral pars orbitalis, pars triangularis, and pars opercularis. No significant results were identified for either participant for conditions of interest.

## Discussion

### Control Participants

A detailed discussion of control results has been reported elsewhere (MacDonald et al., under review), however, brief consideration of the relevant neuroimaging findings is set out below. The majority of whole brain results were identified within right hemisphere regions, confirming previous findings that the right hemisphere can be heavily involved in normal word production processes (Fridriksson et al., 2006a; Mechelli et al., 2007). Focusing on results in language-related regions: repetition suppression effects for only long-term facilitated items were identified in two bilateral areas previously linked to semantic processing (pars triangularis of the inferior frontal gyrus and the posterior inferior temporal gyrus). These findings suggest that long-term repeated naming may have resulted in more efficient retrieval and/or selection of lexical representations for production (pars triangularis; Poldrack et al., 1999; Wagner et al., 2000; Price, 2012; MacDonald et al., under review) and integration of visually derived concepts with their corresponding lexical-semantic representations (inferior temporal gyrus; Koutstaal et al., 2001; Indefrey and Levelt, 2004; Vigneau et al., 2006; Badre and Wagner, 2007; Snyder et al., 2011; MacDonald et al., under review). However, no facilitatory effects were identified in the short-term condition, despite this condition showing the greatest behavioral priming. It could be that priming of these representations has indeed occurred, but was not identified due to insufficient sensitivity of the imaging paradigm, or that effects only become apparent when strengthened by multiple repetitions of stimuli (as in the long-term condition).

While falling outside the scope of discussion in MacDonald et al. (under review), repetition enhancement effects for both facilitated conditions were identified in the left and right precuneus and in the right middle frontal gyrus. These findings outside traditional language-related regions could represent a marker of successful episodic recall (precuneus; Cavanna and Trimble, 2006; Cavanna, 2007) and attention (middle frontal gyrus; Naghavi and Nyberg, 2005).

### Participants with Aphasia

In contrast to the behavioral results for the group of control participants, no improvement was identified in picture naming accuracy for the individual participants with aphasia following short-term facilitation. However, both participants showed improvement in accuracy following multiple repeated attempts at naming several days earlier in the long-term facilitation condition. This finding was stronger for P02 for whom the improvement was over and above that of the unfacilitated-unnamed condition (Mann–Whitney U, *p* = 0.005). For P01 there was not a significant difference between the unfacilitated-unnamed control condition and the long-term facilitated condition, and hence we cannot rule out that for P01 the improvement reflected regression to the mean, given that stimuli were purposefully selected from items that were consistently difficult to name at pretest. For the unfacilitated stimuli that were previously able to be named, as expected under the stressful conditions of the scanner and also consistent with regression to the mean, performance decreased during the scanning session (see **Figure 3**). Consideration of the behavioral results suggests that a single prior attempt at naming by these two individuals with aphasia did not facilitate subsequent naming, or did not facilitate sufficiently to improve accuracy, but that the cumulative effect of multiple attempts was facilitatory.

The only significant neuroimaging result was for P02, with a repetition enhancement effect identified for long-term facilitated items when compared to unfacilitated-named items in the left precuneus. While P01 also showed behavioral benefits for long-term facilitation, no significant neuroimaging results were identified for relevant contrasts, possibly due to a lack of power for this condition. Interestingly, the result for P02 mirrored one of the findings identified for control subjects in the same neural region (left precuneus) and for the equivalent contrast (long-term versus unprimed). A relative increase in neural activation, or repetition enhancement, is often present when some form of additional processing is required (Henson et al., 2000; van Turennout et al., 2003). For our two conditions the same task of overt naming was required and, as such, there should not have been additional processing required specifically for long-term items. However, the conditions differed in two respects: facilitation (several prior exposures for long-term items and no prior exposure for unfacilitated-named items) and previous ability to name (difficult to name for long-term and correctly named for unfacilitated-named). It should be noted that the precuneus is commonly engaged during semantic tasks (Binder et al., 2009), however, this activation has also been interpreted in terms of episodic memory mechanisms (Köhler et al., 2000; Cavanna and Trimble, 2006). We suspect that for P02 an increase in activation for long-term items in this region is more likely related to episodic encoding or object recognition systems being enhanced during subsequent naming of facilitated items with multiple prior exposures, rather than any difference in lexical-semantic processing. Other picture naming studies with aphasic participants have also found increased activity in the precuneus region, with the authors making similar assumptions to the current study with respect to episodic memory mechanisms and further proposing that modulation of activity in regions not traditionally associated with language processing may represent a form of compensatory cortical adaptation (Musso et al., 1999; Fridriksson et al., 2007). In other words, an intact neural region performs strategic compensatory functions enabling completion of, in this case, a linguistic task (Grafman, 2000).

For the long-term condition in controls, we also saw repetition suppression effects in the left IFG (pars orbitalis, pars triangularis, and pars opercularis) when compared to the unprimed condition. However, a subsequent ROI analysis in participants with aphasia in this same region did not reveal any significant results, despite the IFG being spared in both participants. Taken together, these findings demonstrate that while some processing associated with successful word retrieval may be similar across control subjects and individuals with aphasia, facilitation is not necessarily tapping into mechanisms within unimpaired or homologous language-related regions. In this respect our results challenge the assumption that spared left hemisphere language areas and their right hemisphere homologues necessarily support the reorganization of language following stroke (Saur et al., 2006) and reinforce the findings of other studies which conclude that regions not traditionally associated with language processing may contribute significantly to anomic recovery in some individuals with aphasia (Fridriksson et al., 2007; Heath et al., 2013).

### Limitations

The current study could be improved in a number of ways. Only two individuals with aphasia participated, therefore, it is not possible to make claims that may be generalized to the wider aphasic population. A study with a larger series of people with aphasia, after controlling for such factors as symptoms or lesion characteristics, could utilize a whole brain regression analysis to identify whether variation in behavioral facilitation effects relate to variation in brain activity. Additionally, a high percentage of incorrect responses were excluded from the neuroimaging analyses due to the methodological decision to source facilitated conditions for the participants with aphasia from difficult to name items. Therefore, some neuroimaging results were based on only a small number of critical trials and could not be considered due to lack of power (see Supplementary Table S1).

## Conclusion

Even bearing in mind the limitations mentioned above, the findings of the current study have clear implications for the clinical treatment of anomia. Both the control group and the participants with aphasia showed long-term behavioral facilitation effects from repeated picture naming. Although the current study included data from only two individuals with aphasia, our results add further weight to previous evidence that improvement in word retrieval abilities is possible even in chronic aphasia and without direct treatment. However, while the control group showed significant short-term facilitation effects from repeated naming, the aphasic participants did not. In contrast to our original hypothesis, a single prior exposure over a period of minutes did not effectively facilitate naming performance in participants with aphasia. Longer lasting benefits appeared to require multiple exposures, suggesting that the number of times a particular task, or particular items, are presented may be a predictor of treatment outcome. It should be noted though, that the number of repeated naming attempts in the current study involved only six exposures of the same pictorial stimuli across two sessions spanning several days. If a small number of prior naming attempts can result in improvements in behavioral performance, then the impact of any assessment tasks administered prior to beginning a formal treatment regime must be considered. Assessment of anomia, particularly within a cognitive neuropsychological approach, often involves repeated use of the same stimuli across tasks, yet it is generally assumed that assessment has little impact on subsequent naming performance. Our finding of improvement in performance following a limited number of presentations, and in the absence of feedback or correction, emphasizes the power of assessment in a clinical setting, as well as the importance of baselines and control tasks in experimental settings. Moreover, although it is not yet clear that these results would replicate across all people with aphasia, the fact that this task simply required attempts at naming highlights its potential as a straightforward method by which individuals can self-administer treatment.

This study also sought to determine whether any facilitatory effects associated with repeated picture naming act through normal priming mechanisms. We hypothesized that if the mechanisms at work in unimpaired speakers underlie effective anomia treatment, then similar effects would be evident across controls and participants with aphasia in the same language-related neural regions. As expected, neuroimaging results for the control group did identify repetition suppression effects for long-term facilitated items in several regions known to be involved in language processing, mirroring the behavioral improvement for this condition, and possibly reflecting more efficient lexical-semantic processing. In addition to repetition suppression, a repetition enhancement effect was evident in the bilateral precuneus for long-term facilitated items when compared to unprimed items for controls. Conversely, we did not find any neuroimaging results surviving correction in language-related regions for the participants with aphasia. The only significant result identified for one of the participants with aphasia was a repetition enhancement effect outside the language network. Interestingly, this result was identified within the same neural region (left precuneus) and for the equivalent contrast (long-term versus unfacilitated-named items) as in the control group. In line with previous research investigating the role of this brain region, we conclude that an increase in activation for long-term items in the left precuneus is related to episodic encoding or enhancement of object recognition systems, rather than a change in lexical-semantic processing. However, regardless of the source of facilitation, this finding indicates that some individuals with aphasia may engage highly networked regions to support word production processes. Brain areas not traditionally associated with language processing may contribute significantly to recovery in anomia, and it is therefore imperative that, in addition to language, clinicians consider other areas of cognition such as memory and attention in the assessment and treatment of word finding difficulties.

Approaches to word retrieval treatment are not well-understood in terms of underlying neurocognitive mechanisms, however, the findings of the current study add to our understanding of the brain-behavior relationship responsible for positive effects during treatment. This translational knowledge may provide clinicians with some insight into how treatment works in the brain, as well as contribute to the provision of more targeted therapy for individuals with aphasia.

## Conflict of Interest Statement

The authors declare that the research was conducted in the absence of any commercial or financial relationships that could be construed as a potential conflict of interest.

## Footnotes

1 www.fil.ion.ucl.ac.uk/spm

2 http://www.nitrc.org/projects/wfu_pickatlas/

## Supplementary Material

Table S1 **Full whole brain results. MNI coordinates of peak activation from whole brain analyses (*p* < 0.01) for clusters a minimum of 20 contiguous voxels. Automated anatomical labeling software (Tzourio-Mazoyer et al., 2002) was used to identify peak maxima neuroanatomical locations**.
