# Supplementary material for: An fMRI investigation of the effects of attempted naming on word retrieval in aphasia
Source: Front Hum Neurosci. 2015 May 26;9:291. doi: 10.3389/fnhum.2015.00291 (PMC4443028; doi:10.3389/fnhum.2015.00291)
Supplement: Table S1 — Full whole brain results. MNI coordinates of peak activation from whole brain analyses (p < 0.01) for clusters a minimum of 20 contiguous voxels. Automated anatomical labeling software (Tzourio-Mazoyer et al., 2002) was used to identify peak maxima neuroanatomical locations. [file Data_Sheet_1.DOCX]

| P01 | | | | | | P02 | | | | | |
| --- | --- | --- | --- | --- | --- | --- | --- | --- | --- | --- | --- |
| Contrast/Label | Vol | x | y | z | Z | Contrast/Label | Vol | x | y | z | Z |
| LT>UNF-named: | - | - | - | - | - | LT>UNF-named:  Left Precuneus | 91 | -9 | -69 | 39 | 3.50 |
| UNF-named>LT: | - | - | - | - | - | UNF-named>LT: | - | - | - | - | - |
| LT>ST: | - | - | - | - | - | LT>ST2: | - | - | - | - | - |
| ST>LT: | - | - | - | - | - | ST2>LT: | - | - | - | - | - |
| UNF-named>ST: | - | - | - | - | - | UNF-named>ST: | - | - | - | - | - |
| ST>UNF-named: | - | - | - | - | - | ST>UNF-named: | - | - | - | - | - |
| LT>UNF-unnamed:   Left Precuneus | 73 | -12 | -51 | 81 | 3.65 | LT>UNF-unnamed:  Left Precuneus  Left Inferior Parietal  Right Middle Temporal Gyrus  Right Superior Temporal Gyrus  Right Middle Frontal Gyrus (orbital)  Left Cerebellum Crus II | 103  55  21  26  69  44 | -3  -39  63  54  3  -21 | -66  -54  -27  -54  54  -78 | 36  42  -15  21  -3  -36 | 3.60  3.45  3.13  3.04  2.87  2.73 |
| UNF-unnamed>LT:  Cerebellum Vermis VI  Right Inferior Temporal Gyrus | 48  53 | 3  51 | -75  -54 | -9  -12 | 3.58  3.56 | UNF-unnamed>LT:  Left Superior Occipital Gyrus | 34 | -27 | -60 | 24 | 3.28 |
| UNF-named>UNF-unnamed:  Right Postcentral Gyrus  Left Precuneus | 32  33 | 60  -9 | -3  -48 | 30  81 | 2.97  2.91 | UNF-named>UNF-unnamed:  Left Precuneus | 24 | -9 | -63 | 57 | 2.75 |
| UNF-unnamed>UNF-named:  Right Inferior Temporal Gyrus | 31 | 54 | -54 | -15 | 3.45 | UNF-unnamed>UNF-named:  Left Superior Occipital Gyrus  Left Inferior Frontal Gyrus (triangularis)  Cerebellum Vermis I and II | 57  28  21 | -24  -39  -3 | -63  33  -33 | 21  9  -21 | 3.42  3.04  2.94 |
| ST>UNF-unnamed:  Left Anterior Cingulum | 24 | -3 | 48 | 0 | 2.62 | ST>UNF-unnamed:  Right Middle Frontal Gyrus (orbital)  Left Cerebellum Crus II  Right Middle Temporal Gyrus  Right Inferior Parietal | 30  238  24  33 | 36  -9  66  45 | 51  -81  -33  -54 | -9  -30  -3  48 | 3.76  3.17  3.07  3.02 |
| UNF-unnamed>ST:  Right Lingual Gyrus | 70 | 6 | -75 | -9 | 3.25 | UNF-unnamed>ST:  Left Superior Occipital Gyrus | 83 | -24 | -63 | 21 | 3.53 |
